# Efficient synthesis of benzoacridines and indenoquinolines catalyzed by acidic magnetic dendrimer

**DOI:** 10.1038/s41598-024-59212-2

**Published:** 2024-04-16

**Authors:** Mohammad Ali Bodaghifard, Hanieh Allahbakhshi, Rezvan Ahangarani-Farahani

**Affiliations:** 1 Department of Chemistry, Faculty of Science, Arak University, 384817758 Arak, Iran; 2 Institute of Nanosciences and Nanotechnology, Arak University, 384817758 Arak, Iran

**Keywords:** Sustainability, Hybrid nanomaterial, Dendrimer, Catalysis, Acridine, Quinoline, Organic chemistry, Catalysis, Heterogeneous catalysis

## Abstract

A novel solid acid catalyst with recoverability, named as Fe_3_O_4_@SiO_2_@TAD-G2-SO_3_H, was successfully synthesized by immobilizing sulfonic acid groups on triazine dendrimer-modified magnetic nanoparticles. This nanomaterial structure and composition were thoroughly characterized using various analytical techniques, including thermogravimetric analysis (TGA), elemental analysis, Fourier transform infrared spectroscopy (FT-IR), energy-dispersive X-ray spectroscopy (EDX), elemental mapping, acid–base titration, X-ray diffraction (XRD), scanning electron microscopy (SEM), transmission electron microscopy (TEM), and vibrating sample magnetometry (VSM). The acid-decorated magnetic dendrimer was served as a highly effective catalyst for the synthesis of tetrahydrobenzo[*c*]acridin-8(9*H*)-one and benzo[*h*]indeno[1,2-*b*]quinoline-8-one derivatives. The reaction proceeded smoothly under mild conditions through the one-pot condensation of aromatic aldehydes, 1-naphthylamine, and either dimedone or 1,3-indanedione, affording the desired products in high yields ranging from 90 to 96%. The catalyst was easily separated from the reaction mixture by employing a magnetic field, allowing for its recycling up to five times with slight loss in its activity (only 10%). Nearly, quantitative recovery of catalyst (up to 95%) could be obtained from each run. So, this catalyst facilitates the reaction progress and simplifies the purification process. Other remarkable features of this method are operational simplicity, excellent yields, mild condition, and a wide range of substrate applicability.

## Introduction

The development of efficient and environmentally friendly synthesis methods for complex organic molecules is crucial in modern organic chemistry. Traditionally, organic synthesis involved multiple steps, each requiring individual reactions and purification processes. This approach consumed significant amounts of energy and generated substantial waste. multicomponent reactions (MCRs), on the other hand, enable the simultaneous assembly of multiple reactants into complex molecules in a single step. The development and utilization of MCRs has revolutionized the field of organic and medicinal chemistry^1^. The ability to combine multiple starting materials in a one-pot reaction greatly streamlines the synthesis process, minimizes energy consumption and waste generation, saves time and resources, and enhance overall synthetic efficiency. In addition, MCRs often exhibit high atom economy, meaning that a large proportion of the starting materials are incorporated into the final product without generating unnecessary byproducts^2^.

Acridine derivatives have attracted considerable attention for their potential pharmacological activity. These compounds have shown promising results in various areas of medicine, including anticancer, antimalarial, antimicrobial, anti-inflammatory, and calcium antagonist activities^3–8^. Their ability to inhibit DNA (deoxyribonucleic acid) replication and induce apoptosis has made them attractive candidates for the development of novel chemotherapeutic agents^9^.

The wide range of biological activities exhibited by quinoline derivatives has garnered significant interest among organic chemists. Consequently, numerous synthetic pathways have been devised to facilitate the synthesis of quinoline-fused heterocycles. Indenoquinoline derivatives exhibit various pharmacological effects, including antitumor, antimalarial, anti-inflammatory, steroid reductase and acetylcholinesterase inhibitory, and anticancer activities^10–12^. These properties render them highly promising candidates for the development of diverse therapeutic agents. Several synthetic strategies and catalysts, such as succinimide-N-sulfonic acid, tribromomelamine, cellulose-based magnetic nanocomposite, benzyltriethylammonium chloride, tetramethylguanidinium carboxylate ionic liquid, L-Proline, KO_2_ with 18-crown-6 ether, and Cu/MCM-41 have been developed for creating substituted acridine and quinolone derivatives^13–22^. However, many of the existing literature reports involve the use of organic solvents, multiple steps, harsh conditions, and laborious work-up procedures. Therefore, there is a growing demand for more efficient and sustainable methods that minimize the use of hazardous chemicals and simplify experimental procedures.

Dendrimers are unique materials that possess polymer-like properties, characterized by their intricate tree-like branching structures. These structures, combined with their reactive terminal functional groups, branched and regular three-dimensional framework, and internal cavities capable of accommodating targeted ions, have propelled dendrimers to the forefront of various fields such as biomedical research, environmental studies, nanotechnology, supramolecular chemistry, contaminant removal, chemical sensors, catalysis, and drug delivery applications^23^. Compared to traditional polymers, dendrimers exhibit exceptional physical and chemical properties owing to their three-dimensional structures, well-defined shape, uniform size distribution, remarkable flexibility, and multifunctional nature. These highly branched and nano-sized macromolecules offer hundreds of potential binding sites for active species, thus showcasing their immense potential in binding to specific molecules which opens up a world of possibilities for targeted drug delivery, efficient contaminant removal, and enhanced catalytic processes^24–26^.

The advancements in nanotechnology have opened up a world of possibilities for various fields, including medicine, miRNA (microRNA) detection, environmental science, environmental remediation, bio-sensing, and materials engineering. Furthermore, the development of active and intelligent nanoscale systems has paved the way for new materials with enhanced properties and functionalities^27,28^. Magnetic nanoparticles (MNPs) have emerged as versatile supports for active molecules, finding extensive applications in catalysis and pollutant removal^29,30^. In recent years, magnetic nanoparticles have taken center stage as the primary materials for creating dendritic structures, giving rise to magnetic-core dendrimers (MNDs)^31–34^. These nanoparticles can be easily recovered from aqueous solutions using an external magnet, eliminating the need for additional processes like centrifugation. The magnetic core is effectively shielded by a silica shell, which greatly enhances its stability. Moreover, the surface of these particles can be functionalized by attaching diverse active agents through reactions with the silanol groups. This process ensures the protection of the magnetic core and also allows for the incorporation of different functional groups onto the particle surface, thereby expanding its potential applications^28,35–38^.

Developing strategies to control the arrangement of different functional groups and active sites on the surface of nanoparticles remains a crucial task for effective catalytic applications^39–42^. One approach to enhance this control is to introduce a polymer or dendritic shell onto the nanoparticle surface. This not only improves the placement of functional groups but also enhances the dispersion of the nanoparticles in organic solvents, which further enhances their catalytic performance. Notably, star-like dendritic structures exhibit exceptional properties, boasting a multitude of functional groups that significantly increase the number of active sites on the nanoparticle surface^43^. The advantages of using dendritic magnetic nanostructures as catalysts are numerous. Their high surface area and surface functionality provide active sites for catalytic reactions. Additionally, their magnetic properties allow for easy separation, recyclability, simplifies the purification process and saves us from the tedious task of removing impurities. These nanostructures have shown exceptional catalytic activity and selectivity in various organic transformations, making them promising candidates for enhancing reaction efficiency^44–48^.

**Figure 1:**
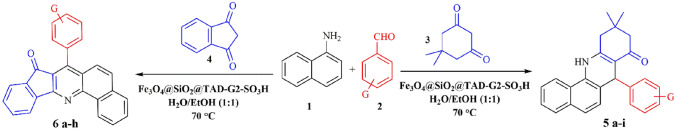
synthesis of benzo[*c*]acridin-8(9*H*)-ones and benzo[*h*]indeno[1,2-*b*] quinolin-8-ones in the presence of Fe_3_O_4_@SiO_2_@TAD-G2-SO_3_H.

Considering the unique properties of both magnetic nanoparticles (MNPs) and dendrimers, this research focuses on the preparation of dendrimer-decorated magnetic nanoparticles (Fe_3_O_4_@SiO_2_@Den-SO_3_H) and their application in the one-pot synthesis of benzo[*c*]acridine-8(9*H*)-one and benzo[*h*]indeno[1,2‐*b*]quinoline‐8‐one derivatives, two structurally diverse and biologically active compounds of interest (Fig. 1).

## Experimental

Cyanuric chloride, tetrabutylammonium bromide, ethanolamine, chlorosulfonic acid, dioxane were commercially supplied by Merck and Acros companies. FeCl_3_.(H_2_O)_6_, FeCl_2_.(H_2_O)_4_, KOH, NaOH, HCl, and ethanol were commercially supplied by neutron chemical company (Iran). All the materials and reagents were used without any further purification. Fourier-transform infrared spectroscopy (FT-IR) measurements were conducted using a KBr pellet and an Alpha Bruker Fourier transform (FT-IR) spectrophotometer, covering the range of 400–4000 cm^−1^. The crystallinity of the nanomaterials was assessed using. A Philips Xpert X-ray powder diffraction (XRD) instrument, employing Cu-Kα radiation with a wavelength of λ = 0.1545 was applicated to evaluate the crystallinity of nanomaterials. To examine the surface morphology and elemental distribution, a MIRA 3-XMU field emission scanning electron microscope (FE-SEM) equipped with energy dispersive X-ray analysis (EDX) was employed. Thermogravimetric analysis (TGA) was performed on a Mettler TA4000 system under a nitrogen atmosphere, within the temperature range of 25–800 °C, with a heating rate of 15 °C/min. The magnetic properties of the nanoparticles were investigated using a 730 vibrating sample magnetometer (VSM) at room temperature.

### Preparation of Fe_3_O_4_@SiO_2_-TzCl_2_ nanoparticles

The Fe_3_O_4_@SiO_2_-TzCl_2_ nanoparticles were prepared based on our previous report^34^.

### Preparation of first generation dendritic magnetic nanoparticles (Fe_3_O_4_@SiO_2_@TAD-G1).

The Fe_3_O_4_@SiO_2_-TzCl_2_ (1 g) was dispersed in 20 mL of dioxane for a duration of 20 min. Subsequently, K_2_CO_3_ (5 mmol, 0.7 g) and ethanolamine (10 mmol, 0.7 g) were introduced to the mixture and subjected to reflux for a period of 24 h. The resulting solid was collected using an external magnet, rinsed with ethanol, and then dried under vacuum at a temperature of 50 °C.

### Synthesis of second generation dendritic magnetic nanoparticles (Fe_3_O_4_@SiO_2_@TAD-G2).

Fe_3_O_4_@SiO_2_@TAD-G1 (1 g) was poured into a round-bottom flask and dispersed in dichloromethane (50 mL) for 10 min. Then, Cyanuric chloride (10 mmol, 1.8 g), tetrabutylammonium bromide (TBAB) (0.001 g), and 1 mL of NaOH (10 M) were added to the suspension and stirred for 2 h at 5 °C in an ice water bath. Afterward, the mixture was stirred at room temperature for 24 h. The resulting black precipitate was isolated using an external magnet, washed with dichloromethane, and dried. Next, the obtained particles (1 g) were mixed with K_2_CO_3_ (10 mmol, 1.4 g) and ethanolamine (20 mmol, 1.2 g) in dioxane (50 mL). The suspension was refluxed for 24 h. The resulting precipitate was isolated, washed with ethanol, and dried under vacuum at 50 °C.

### Preparation of acid-decorated dendritic nanoparticles (Fe_3_O_4_@SiO_2_@TAD-G2-SO_3_H)

Fe_3_O_4_@SiO_2_@TAD-G2 (1 g) was dispersed in 40 mL of dichloromethane and stirred for 20 min. Subsequently, a solution of chlorosulfonic acid (2 mL) in 5 mL of dichloromethane was meticulously prepared and slowly added to the mixture over the course of 1 h. The resulting solid was collected using an external magnet, thoroughly washed with dichloromethane, and subsequently dried under vacuum at 50 °C.

### Acidity of Fe_3_O_4_@SiO_2_@TAD-G2-SO_3_H

The concentration of sulfonic acid groups was determined by quantitatively estimating it using back titration with HCl (0.01 N). Initially, 2 mL of KOH (0.01 N) was added to 0.02 g of the Fe_3_O_4_@SiO_2_@TAD-G2-SO_3_H nanoparticles, and the mixture was stirred for a duration of 30 min. Subsequently, the catalyst was separated and thoroughly washed with deionized water. To determine the excess amount of KOH, it was titrated with HCl (0.01 N) in the presence of phenolphthalein as an indicator. To ensure precision, three separate titrations were conducted, and the average value was calculated to ascertain the acid content of Fe_3_O_4_@SiO_2_@TAD-G2-SO_3_H. The findings revealed that this nanostructure possessed an acid content of 2.5 mmol g^−1^.

### General procedure for the synthesis of benzo[*c*]acridin-8(9*H*)-ones (5a–i):

In a 25 mL round bottom flask, an aromatic aldehyde (1 mmol), dimedone (1 mmol, 0.14 g), and 1-naphthylamine (1 mmol, 0.143 g) were combined in 5 ml of ethanol/water (1:1). This mixture was stirred in the presence of Fe_3_O_4_@SiO_2_@TAD-G2-SO_3_H (0.03 g) at a temperature of 70 °C. To monitor the progress of the reaction, thin-layer chromatography (TLC) with a solvent mixture of n-hexane and ethyl acetate (2:1) was used. Once the reaction was completed, the catalyst separated from the reaction medium using a magnet. Next, the solvent was evaporated and the product recrystallized using ethanol. Finally, the product was dried in an oven at 50 °C.

### General procedure for the synthesis of benzo[*h*]indeno[1,2-*b*]quinolin-8-ones (6a–h):

In a 25 mL round bottom flask, an aromatic aldehyde (1 mmol), 1,3-indanedione (1 mmol, 0.146 g), 1-naphthylamine (1 mmol, 0.143 g), and Fe_3_O_4_@SiO_2_@TAD-G2-SO_3_H (0.03 g) was combined in 5 mL of a 1:1 mixture of ethanol/water. The mixture was stirred in the presence of 5 mL of a 1:1 mixture of ethanol and water at a temperature of 70 °C. Thin-layer chromatography (TLC) with a solvent mixture of n-hexane and ethyl acetate in a ratio of 3:1 was used to monitor the progress of the reaction. Once the reaction was completed, the catalyst was separated from the reaction medium using a magnet, and the solvent was evaporated. Finally, the product was recrystallized using ethanol and dried in an oven at 50 °C.

### Spectral data for selected products

#### 7-(4-chlorophenyl)-10,10-dimethyl-7,10,11,12-tetrahydrobenzo[*c*]acridin-8(9*H*)-one (5b)

In a 25 mL round bottom flask, 4-chlorobenzaldehyde (1 mmol, 0.141 g), 1,3-indanedione (1 mmol, 0.146 g), 1-naphthylamine (1 mmol, 0.143 g), and Fe_3_O_4_@SiO_2_@TAD-G2-SO_3_H (0.03 g) was combined in 5 mL of a 1:1 mixture of ethanol/water. The mixture was stirred at a temperature of 70 °C. Once the reaction was completed, the catalyst was separated from the reaction medium using a magnet, and the solvent was evaporated. Finally, the product was recrystallized using ethanol and dried in an oven at 50 °C. Melting point: 268–270 °C. IR: 3335 (NH), 2957 (CH_Ar_), 2930 (CH_Al_), 1606 (C = O), 1573 (C = C), 1492 (C = C), 1261 (C–N), 847 cm^−1^. ^1^HNMR (300 MHz, DMSO-*d*_*6*_): δ = 0.95 (s, 3H, CH_3_), 1.05 (s, 3H, CH_3_), 2.04 (d, J = 16.0 Hz, 1H, CH_2_), 2.20 (d, J = 16.0 Hz, 1H, CH_2_), 2.60 (d, J = 16.8 Hz, 1H, CH_2_), 2.71 (d, J = 16.8 Hz, 1H, CH_2_), 4.99 (s, 1H, CH), 6.83–7.52 (m, 10H, ArH), 9.29 (s, 1H, NH) ppm.

#### 7-(4-nitrophenyl)-10,10-dimethyl-7,10,11,12-tetrahydrobenzo[*c*]acridin-8(9*H*)-one (5d)

IR: 3318 (NH), 2957 (CH_Ar_), 2870 (CH_Al_), 1597 (C = O), 1570 (C = C), 1518 (-NO_2_), 1491 (C = C), 1370, 1344 (-NO_2_), 1261 (C–N), 857 ${{\text{cm}}}^{-1}$; ^1^HNMR (300 MHz, DMSO-d6): δ = 0.97 (s, 3H, CH_3_), 1.08 (s, 3H, CH_3_), 2.04 (d, J = 16.4 Hz,1H, CH_2_), 2.22 (d, J = 16.4 Hz, 1H, CH_2_), 2.70 (d, J = 16.8 Hz, 1H, CH_2_), 2.77 (d, J = 16.8 Hz,1H, CH_2_) 5.18 (s, 1H, CH), 7.55–8.07 (m, 11H, ArH), 9.41 (s, 1H, NH) ppm.

#### 7-(3-nitrophenyl)-10,10-dimethyl-7,10,11,12-tetrahydrobenzo[*c*]acridin-8(9*H*)-one (5e)

IR: 3312 (NH)(CH_Ar_), 2955 (CH_Ar_), 2870 (CH_Al_), 1589 (C = O), 1527 (-NO_2_), 1494 (C = C), 1388, 1347(-NO_2_), 1261 (C–N), 807, 756, 729,${{\text{cm}}}^{-1}$; ^1^HNMR (300 MHz, DMSO-d6): δ = 0.97(s, 3H, CH_3_), 1.08 (s, 3H, CH_3_), 2.07 (d, J = 16.4 Hz, 1H, CH_2_), 2.22 (d, J = 16.4 Hz, 1H, CH_2_), 2.27 (d, J = 16.8 Hz, 1H, CH_2_), 2.75 (d, J = 16.8 Hz, 1H, CH_2_) 5.42 (s, 1H, CH), 7.69–8.07 (m, 10H, ArH), 9.41 (s, 1H, NH) ppm.

#### 7-(3-hydroxyphenyl)-10,10-dimethyl-7,10,11,12-tetrahydrobenzo[*c*]acridin-8(9*H*)-one (5h)

IR: 3395 (NH), 3298 (OH), 2957 (CH_Ar_), 2928 (CH_Al_), 1606 (C = O), 1555 (C = C), 1487 (C = C), 1256 (C–N), 761,699,${{\text{cm}}}^{-1}$; ^1^HNMR (300 MHz, DMSO-d6): δ = 0.94 (s, 3H, CH_3_), 1.05 (s, 3H, CH_3_), 2.04 (d, J = 16.0 Hz, 1H, CH_2_), 2.22(d, J = 16.0 Hz, 1H, CH_2_), 2.58 (d, J = 16.8 Hz, 1H, CH_2_), 2.70 (d, J = 16.8 Hz, 1H, CH_2_) 4.86 (s, 1H, CH), 6.39–6.97 (m, 10H, ArH), ), 9.05 (s, 1H, OH), 9.22 (s, 1H, NH) ppm.

#### 7-(2,4-dichlorophenyl)-10,10-dimethyl-7,10,11,12-tetrahydrobenzo[*c*]acridin-8(9*H*)-one (5i)

IR: 3324 (NH), 3067 and 2956 (CH_Ar_), 2870 (CH_Al_), 1607 (C = O), 1588 (C = C), 1496 (C = C), 1259 (C–N), 861, 566,${{\text{cm}}}^{-1}$; ^1^HNMR (300 MHz, DMSO-d6): δ = 0.97 (s, 3H, CH_3_), 1.03 (s, 3H, CH_3_), 2.00 (d, J = 16.0 Hz, 1H, CH_2_), 2.17 (d, J = 16.0 Hz, 1H, CH_2_), 2.22 (d, J = 16.4 Hz, 1H, CH_2_), 2.70 (d, J = 16.4 Hz,1H, CH_2_) 5.48 (s, 1H, CH), 7.14–7.56 (m, 9H, ArH), 9.22 (s, 1H, NH) ppm.

#### 7-(4-Chlorophenyl)-8*H*-benzo[*h*]indeno[1,2-*b*]quinolin-8-one (6b)

IR: 3060 (CH_Ar_), 2923 (CH_Ar_), 1710 (C = O), 1603 (C = N), 1576 and 1484 (C = C), 1337 (C–N), 840 cm^−1^. ^1^HNMR (300 MHz, DMSO-*d*_*6*_): δ = 7.45–8.07 (m, 14H, ArH) ppm.

#### 7-(3-Nitrophenyl)-8*H*-benzo[*h*]indeno[1,2-*b*]quinolin-8-one (6e)

IR: 3060 (CH_Ar_), 2923 (CH_Ar_), 1710 (C = O), 1656 (C = C), 1613 (C = N), 1527 (C = C), 1345 (C–N), 759, 712 cm^−1^. ^1^HNMR (300 MHz, DMSO-*d*_*6*_): δ = 7.36–8.08 (m, 14H, ArH) ppm.

#### 7-(4-Nitrophenyl)-8*H*-benzo[*h*]indeno[1,2-*b*]quinolin-8-one (6f)

IR: 3057 (CH_Ar_), 1712 (C = O), 1662 and 1580 (C = C), 1613 (C = N), 1524 (NO_2_), 1394, 1344 (NO_2_), 1264 (C–N), 859 cm^−1^. ^1^HNMR (300 MHz, DMSO-*d*_*6*_): δ = 7.25–7.96 (m, 14H, ArH) ppm.

#### 7-(3-Hydroxyphenyl)-8*H*-benzo[*h*]indeno[1,2-*b*]quinolin-8-one (6g)

IR: 3406 (OH), 3051 and 2955 (CH_Ar_), 1707 (C = O), 1608 (C = N), 1574 (C = C), 1324 (C–N), 753, 698 cm^−1^. ^1^HNMR (300 MHz, DMSO-*d*_*6*_): δ = 7.35–8.04 (m, 14H, ArH), 9.36 (s, 1H, OH) ppm.

#### 7-(2,4-Dichlorophenyl)-8*H*-benzo[*h*]indeno[1,2-*b*]quinolin-8-one (6h)

IR: 3058 (CH_Ar_), 1715 (C = O), 1607 (C = N), 1654 and 1576 (C = C), 1469, 1338, 851, 569 cm^−1^. ^1^HNMR (300 MHz, DMSO-*d*_*6*_): δ = 6.82–7.56 (m, 13H, ArH) ppm.

## Results and discussion

**Figure 2:**
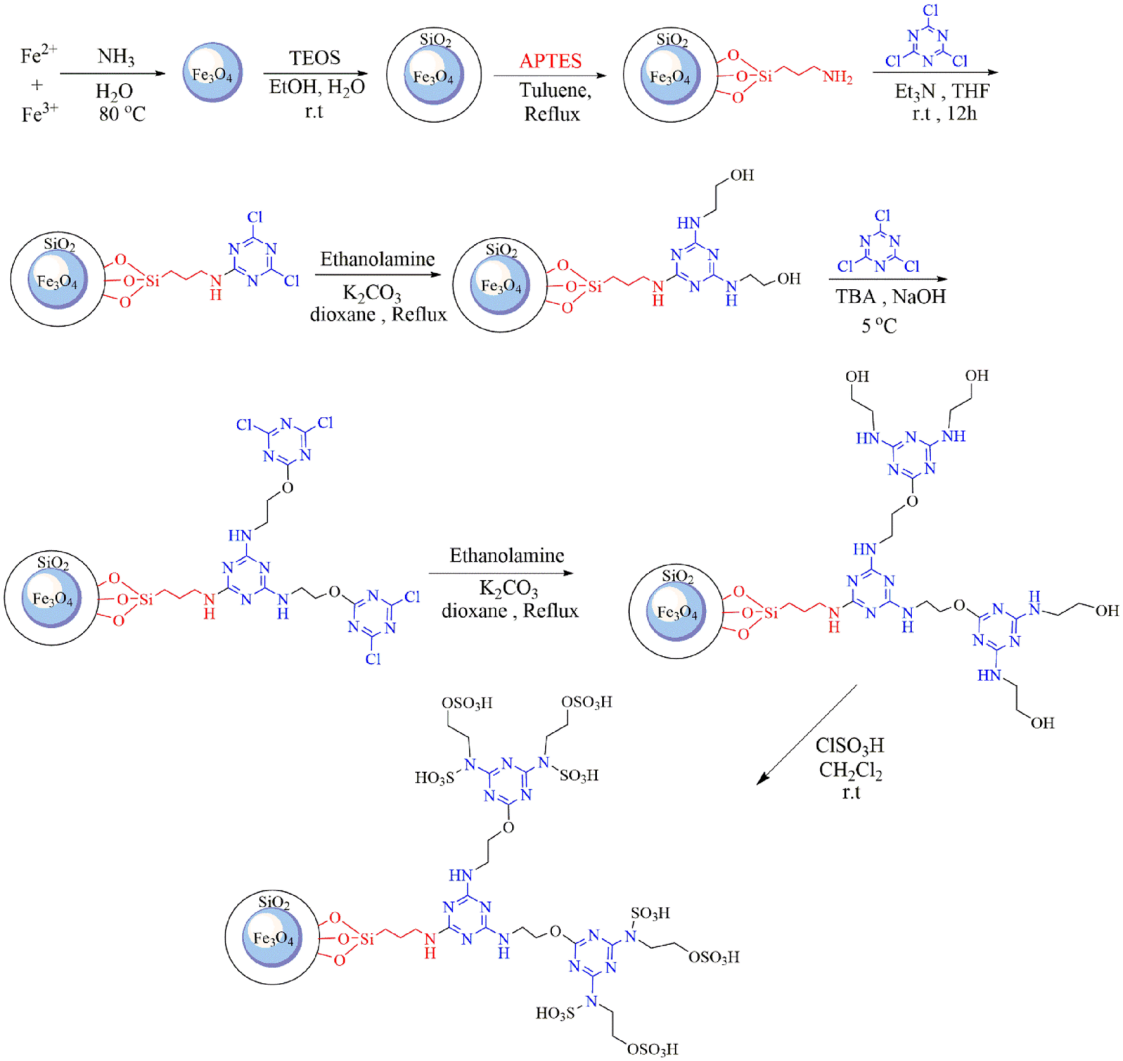
Synthetic pathway for construction of dendrimer-coated magnetite nanoparticles (Fe_3_O_4_@SiO_2_@TAD-G2-SO_3_H).

In this study, sequential reactions were employed to introduce dendrimers onto solid supports (divergent strategy). This was achieved by attaching a linker onto the surface of magnetic nanoparticles and the triazine-based poly-aminoethanol (TAD) dendrimers was successfully grown on the surface of silica-coated magnetite nanoparticles via sequential reactions (Fe_3_O_4_@SiO_2_@TAD-G2-SO_3_H, Fig. 2). Initially, magnetite (Fe_3_O_4_) nanoparticles were easily prepared via the chemical co-precipitation of Fe^2+^ and Fe^3+^ ions in basic solution. Next, a layer of silica was placed on the magnetic iron nanoparticles, via Stober method, to prevent their aggregation and oxidation. Then, the (3-aminopropyl)triethoxysilane (APTMS) as a linker was grafted to the surface of Fe_3_O_4_@SiO_2_ particles under nitrogen atmosphere to produce the Fe_3_O_4_@SiO_2_@PrNH_2_ nanostructure^49^. In the subsequent stages, TAD dendrons (functional arms) up to generation two (G2) were constructed through a sequential and progressive nucleophilic aromatic substitution of ethanolamine on the aromatic triazine ring^34,50^. Ultimately, the nanoparticles were decorated with acidic groups through a reaction of active –NH and –OH groups with chlorosulfonic acid.

**Figure 3:**
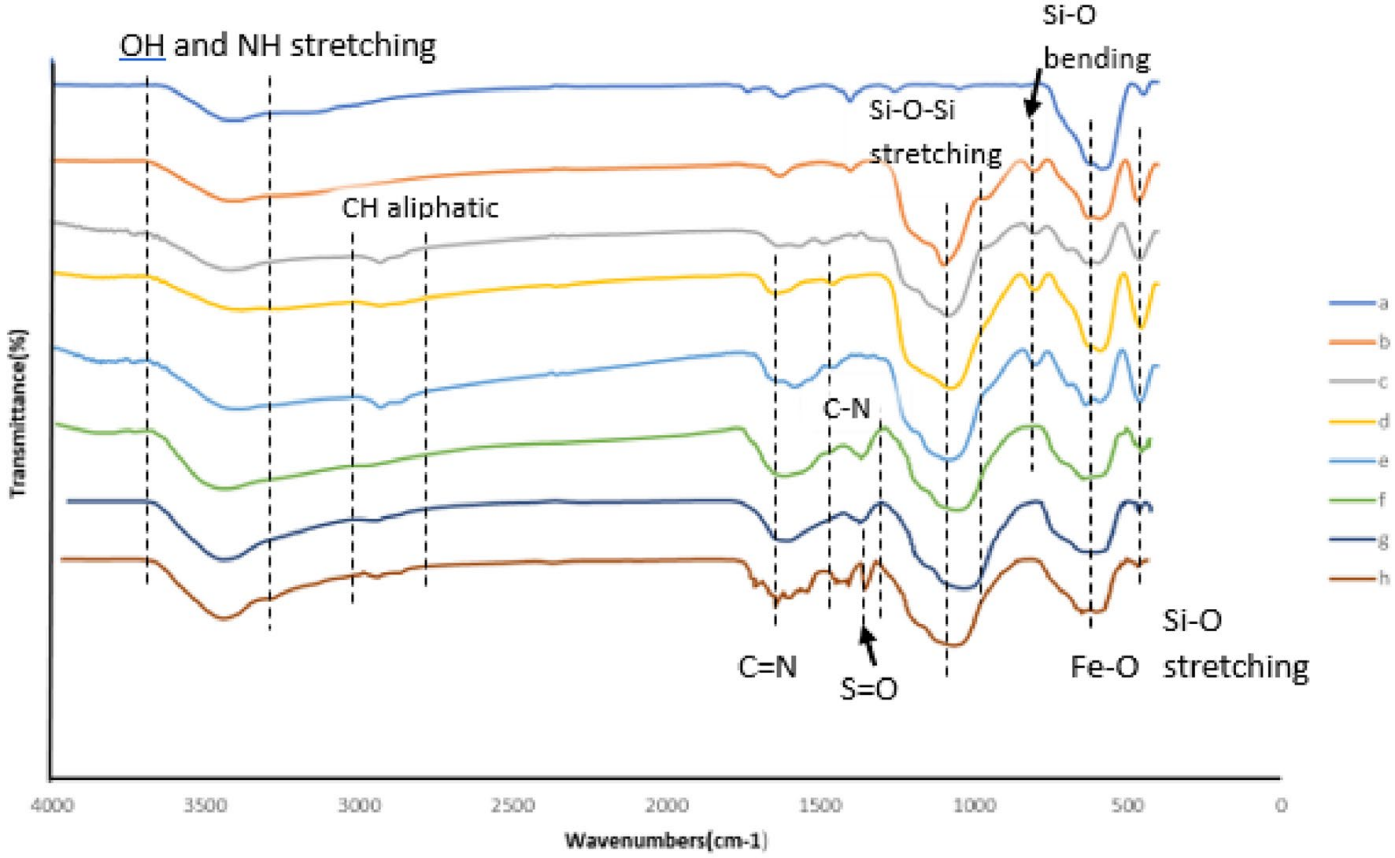
FT-IR spectra of Fe_3_O_4_ (**a**), Fe_3_O_4_@SiO_2_ (**b**), Fe_3_O_4_@SiO_2_ -PrNH_2_ (**c**), Fe_3_O_4_@SiO_2_-TzCl_2_ (**d**), Fe_3_O_4_@SiO_2_@TAD-G1 (**e**), Fe_3_O_4_@SiO_2_ -Tz-BEA-TzCl_4_ (**f**), Fe_3_O_4_@SiO_2_@TAD-G2 (**g**), Fe_3_O_4_@SiO_2_@TAD-G2-SO_3_H (**h**).

Figure 3, shows the FT-IR spectra of Fe_3_O_4_, Fe_3_O_4_@SiO_2_, Fe_3_O_4_@SiO_2_ -PrNH_2_, Fe_3_O_4_@SiO_2_-TzCl_2_, Fe_3_O_4_@SiO_2_@TAD-G1, Fe_3_O_4_@SiO_2_-Tz-BEA-TzCl_4_, Fe_3_O_4_@SiO_2_@TAD-G2, Fe_3_O_4_@SiO_2_@TAD-G2-SO_3_H in the wavenumber range of 400–4000 cm^−1^. The FT-IR spectrum of the bare magnetic Fe_3_O_4_ nanoparticles exhibited the characteristic Fe–O absorption band in the range of 580 cm^−1^ (Fig. 3a). The bands observed at 1101, 900–800, and 450 cm^−1^, correspond to asymmetric stretching, symmetric stretching, in-plane bending of the Si-O-Si and Si–O bonds, respectively (Fe_3_O_4_@SiO_2_ spectrum, Fig. 3b). The band in the region of 1300 cm^−1^ is related to the stretching bond S = O (Fig. 3h). The broad band in the range 3200–3500 cm^−1^ appertained to the stretching vibration mode of Si–OH bonds and the weak band at 1630 cm^−1^ related to the twisting vibration mode of H–O-H adsorbed in the silica layer. The presence of alkyl groups has been corroborated by the weak symmetric and asymmetric stretching vibrations at 2980 and 2895 cm^−1^ (Fig. 3c–h). Also, the bands corresponding to C = N, C = C, and C–N appeared at 1350–1600 cm^−1^ (Fig. 3d–h)^34,51^. The N–H bending vibration is overlapped with O–H vibration. Therefore, these results confirm the successful bonding of different functional groups at each fabrication step.

**Figure 4:**
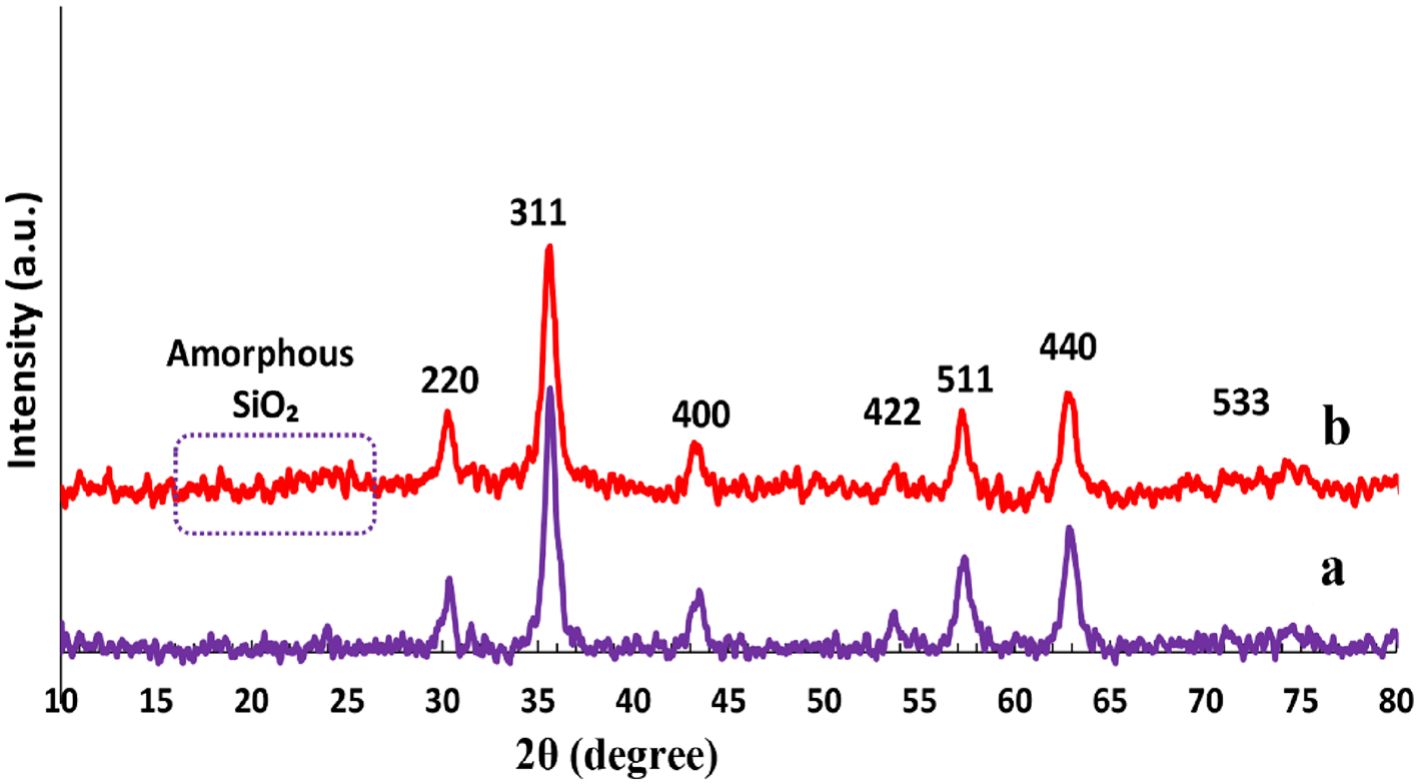
XRD patterns of Fe_3_O_4_ (**a**), JCPDS file No. 19–0629), and Fe_3_O_4_@SiO_2_@TAD-G2-SO_3_H (**b**).

The crystallinity of Fe_3_O_4_ (a), and Fe_3_O_4_@SiO_2_@TAD-G2-SO_3_H (b) was analyzed using X-ray powder diffraction patterns (Fig. 4). By comparing the XRD patterns of Fe_3_O_4_ and Fe_3_O_4_@SiO_2_@TAD-G2-SO_3_H, it can be confirmed that the ferrite core remains stable without undergoing any phase changes during the functionalization process^52^. The XRD patterns of the prepared Fe_3_O_4_@SiO_2_@TAD-G2-SO_3_H show distinct diffraction peaks at 2*θ* = 30.2°, 35.4°, 43.7°, 56.6°, and 63.0°, corresponding to the (220), (311), (400), (422), (511), and (440) Miller planes of Fe_3_O_4_ nanoparticles. These XRD patterns confirm that the material structure has a cubic spinel phase (Fig. 4a,b) and match with the standard Fe_3_O_4_ sample (JCPDS file No. 19–0629)^53,54^. The presence of the SiO_2_ layer is confirmed by the appearance of a broad peak at 2*θ* = 18° to 25° (Fig. 4b). To determine the average crystallite size of Fe_3_O_4_@SiO_2_@TAD-G2-SO_3_H, the Scherrer equation (*D* = *Kλ/βcosθ*) was employed. In this equation, *λ* is the x-ray Cu wavelength, *β* is the width of the X-ray peak on the 2*θ* axis which measured as the line broadening at half the maximum intensity, *θ* is Bragg angle, and *K* is the so-called Scherrer constant. The crystallite size calculated from the width of the peak at 2*θ* = 35.4° (311), is approximately 102 nm^41^.

**Figure 5:**
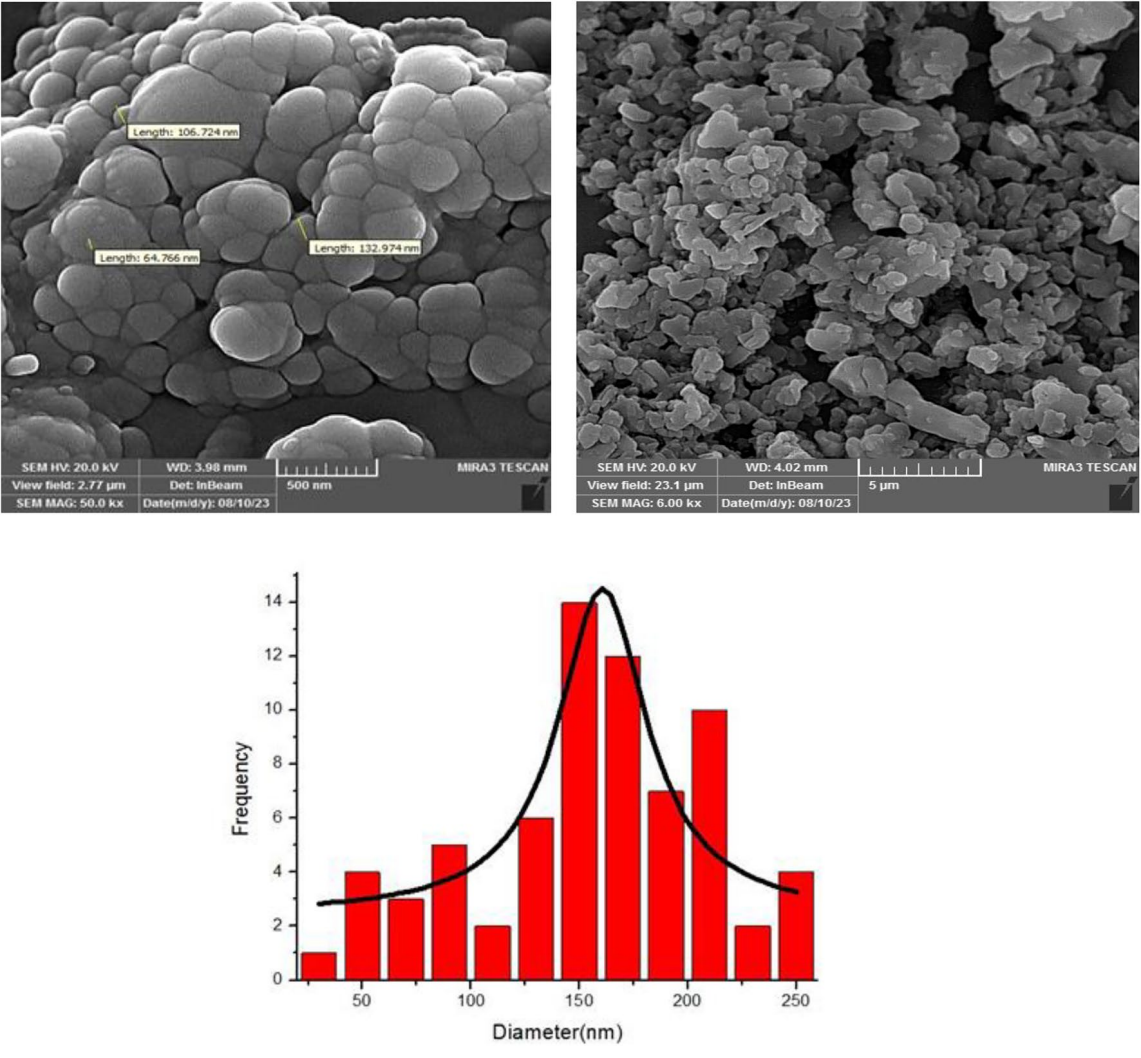
The FE-SEM images and histogram for particles size of Fe_3_O_4_@SiO_2_@TAD-G2-SO_3_H.

The size and morphology of Fe_3_O_4_@SiO_2_@TAD-G2-SO_3_H are investigated by field emission scanning electron microscopy (FE-SEM). As shown in Fig. 5, the Fe_3_O_4_@SiO_2_@TAD-G2-SO_3_H nanostructure possesses nearly spherical morphology with an average size of 100–130 nm.

**Figure 6:**
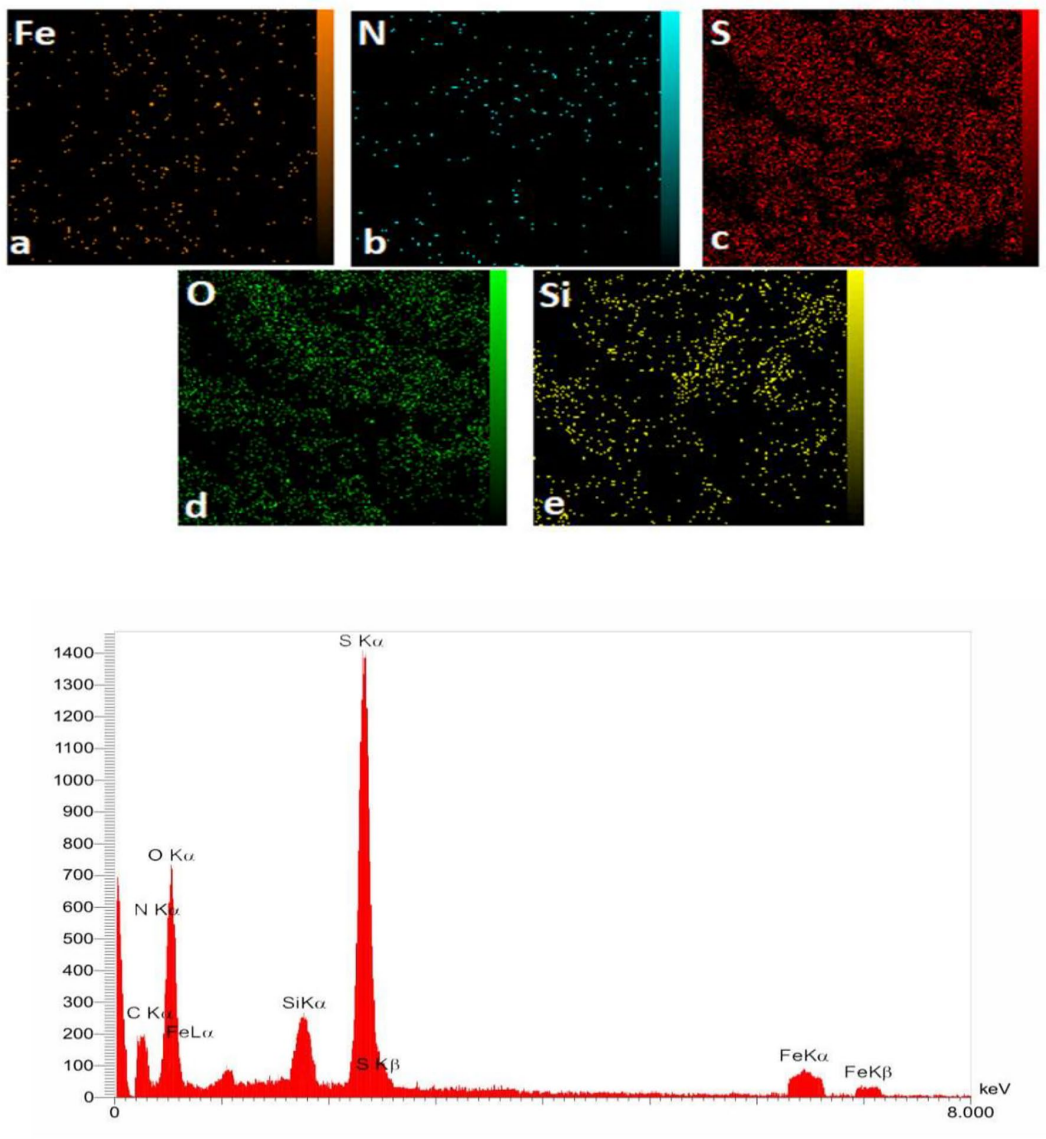
EDX spectrum and EDX and map analysis for Fe_3_O_4_@SiO_2_@TAD-G2-SO_3_H.

The results obtained from X-ray energy diffraction spectroscopy (EDX) of Fe_3_O_4_@SiO_2_@TAD-G2-SO_3_H magnetic nanoparticles (Fig. 6) confirm the presence of elements S, C, N, Si, O, Fe in the hybrid nanostructure. The higher intensity of the peak corresponding to the elements Si and S compared to the Fe peak indicates that the Fe_3_O_4_ nanoparticles have been covered by SiO_2_ layer and –SO_3_H functionalities on the surface of the nanostructure. The terminal ends of the catalyst have a lot of –SO_3_H groups, causing the sulfur have a higher peak intensity. These results provide evidence for the successful synthesis of the core–shell nanostructure (Fe_3_O_4_@SiO_2_@TAD-G2-SO_3_H). Moreover, the EDX map analysis reveals a good distribution of various elements including Fe (a), N (c), S (d), O (e), and Si (f) throughout the nanostructure.

**Figure 7:**
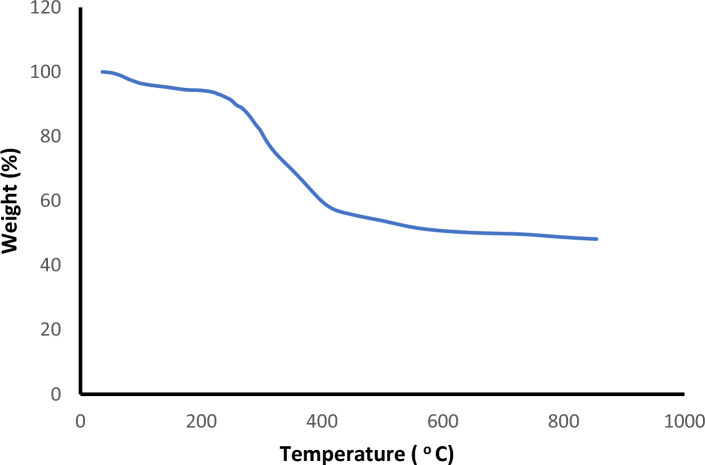
TGA analyses of Fe_3_O_4_@SiO_2_@TAD-G2-SO_3_H.

Thermal gravimetric studies were conducted within the temperature range of 25–800 °C under a N_2_ atmosphere, to evaluate the thermal stability of Fe_3_O_4_@SiO_2_@TAD-G2-SO_3_H (Fig. 7). A weight loss of approximately 4–6% is observed in the temperature range of 25 to 250 °C, which is attributed to the loss of residual solvent and water on the sample surface (physically absorbed) process. Furthermore, a significant weight loss of approximately 40% was observed in 250–650 °C, which can be attributed to the degradation of dendrimer branches and organic moieties, followed by a phase change in the silica nanoparticle groups. The remaining weight (˃650 °C) is attributed to inorganic support which was not decomposed.

**Figure 8:**
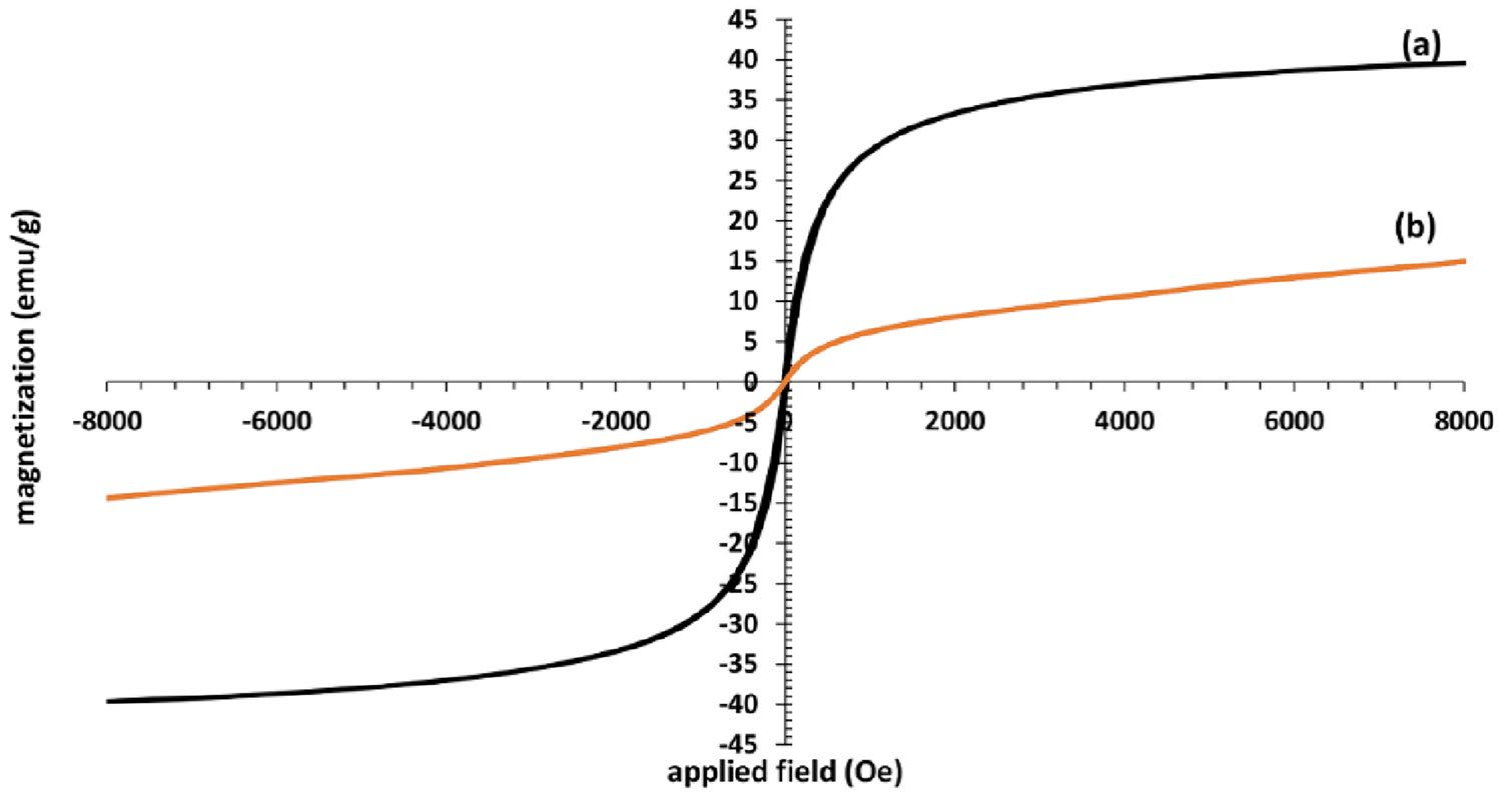
VSM analysis of Fe_3_O_4_ nanoparticles (**a**), and Fe_3_O_4_@SiO_2_@TAD-G2-SO_3_H nanostructure (**b**).

The magnetization curves of Fe_3_O_4_ (a), Fe_3_O_4_@SiO_2_@TAD-G2-SO_3_H (b) were measured using the VSM technique at room temperature, within the range of ± 8000 Oe (Fig. 8). The *S*-like magnetization curves, the coincidence of the hysteresis loop, and the low remanence and coercivity confirm the superparamagnetic properties of these hybrid materials. As seen in Fig. 6, the saturation level for the Fe_3_O_4_ sample is 40 emu g^−1^, While it is 15 emu g^−1^ for Fe_3_O_4_@SiO_2_@TAD-G2-SO_3_H nanostructure. This decrease in magnetic property indicates the presence of a surface coating on the Fe_3_O_4_ nanoparticles, confirming the core–shell structure and the grafting of the dendritic branches to the magnetic nanoparticles.

After the characterization of the Fe_3_O_4_@SiO_2_@TAD-G2-SO_3_H catalyst, its catalytic activity was evaluated for the synthesis of benzo[*c*]acridin-8(9*H*)-one derivatives (Fig. 1). For this purpose, the reaction of 4-chlorobenzaldehyde (1 mmol), dimedone (1 mmol) and 1-naphthylamine (1 mmol) was used as a model and conducted under different reaction parameters including the catalyst amount, solvent, and temperature (Table 1). The best result was achieved when the reaction took place in the presence of 30 mg of the catalyst, in a polar solvent mixture of H_2_O/EtOH (1:1), and at a temperature of 70 °C (Table 1, entry 9). In continuation, the catalytic activity of Fe_3_O_4_@SiO_2_@TAD-G2-SO_3_H catalyst was evaluated for the synthesis of benzo[*h*]indeno[1,2-*b*]quinolin-8-ones. Interestingly, the best result was achieved in H_2_O/EtOH (1:1) at 70 °C, using 30 mg of the catalyst. After obtaining the best conditions for the reaction, various arylaldehydes including electron-withdrawing and electron-releasing groups were subjected to the reactions. All substituted arylaldehydes effectively reacted and afforded the corresponding products in excellent yields without any side product (Table 2).

**Table 1:** Optimization of the conditions for synthesis of benzo[*c*]acridin-8(9*H*)-one derivatives.


Entry	Solvent	Catalyst(mg)	Condition	Time	Yield (%)
1	Solvent free	20	Reflux	10 h	52
2	EtOH	20	Reflux	10 h	70
3	H_2_O	20	Reflux	10 h	60
4	H_2_O	20	50 °C	10 h	62
5	H_2_O	20	70 °C	10 h	65
6	H_2_O/EtOH (1:1)	20	50 °C	6 h	80
7	H_2_O/EtOH (1:1)	20	Reflux	6 h	75
8	H_2_O/EtOH (1:1)	20	70 °C	6 h	90
9	H_2_O/EtOH (1:1)	30	70 °C	3 h	95
10	H_2_O/EtOH (1:1)	50	70 °C	3 h	95

**Table 2:** Synthesis of benzo[*c*]acridin-8(9*H*)-one and benzo[h]indeno[1,2-*b*]quinolin-8-one derivatives using Fe_3_O_4_@SiO_2_@TAD-G2-SO_3_H.


m.p. (reported,**°C**) ^[Ref]^	m.p. (obtained,°C)	Yield(%)	product	R	Entry
258–260 ^18^	260–262	92	**5a**	C_6_H_5_	1
268–270 ^14^	268–270	95	**5b**	4-Cl-C_6_H_4_	2
280–282 ^18^	277–279	93	**5c**	4-Br-C_6_H_4_	3
267–269 ^14^	260–262	94	**5d**	4-NO_2_-C_6_H_4_	4
267–269 ^15^	255–258	92	**5e**	3-NO_2_-C_6_H_4_	5
259–261 ^18^	256–258	91	**5f**	4-CH_3_O-C_6_H_4_	6
210–212 ^18^	214–215	90	**5g**	4-CH_3_-C_6_H_4_	7
218–220 ^14^	221–223	91	**5 h**	3-OH-C_6_H_4_	8
280–282 ^15^	275–277	95	**5i**	2,4-Cl_2_-C_6_H_3_	9
200–203 ^19^	204–206	93	**6a**	C_6_H_5_	10
259–261 ^16^	255–257	96	**6b**	4-Cl-C_6_H_4_	11
240–242 ^16^	250–252	92	**6c**	4-CH_3_-C_6_H_4_	12
230–233 ^14^	234–235	90	**6d**	4-CH_3_O-C_6_H_4_	13
230–233 ^17^	228–230	93	**6e**	3-NO_2_-C_6_H_4_	14
222–224 ^17^	225–227	95	**6f.**	4-NO_2_-C_6_H_4_	15
328–329 ^16^	˃ 300	91	**6g**	3-OH-C_6_H_4_	16
212–215 ^17^	215–217	94	**6h**	2,4-Cl_2_-C_6_H_3_	17

**Figure 9:**
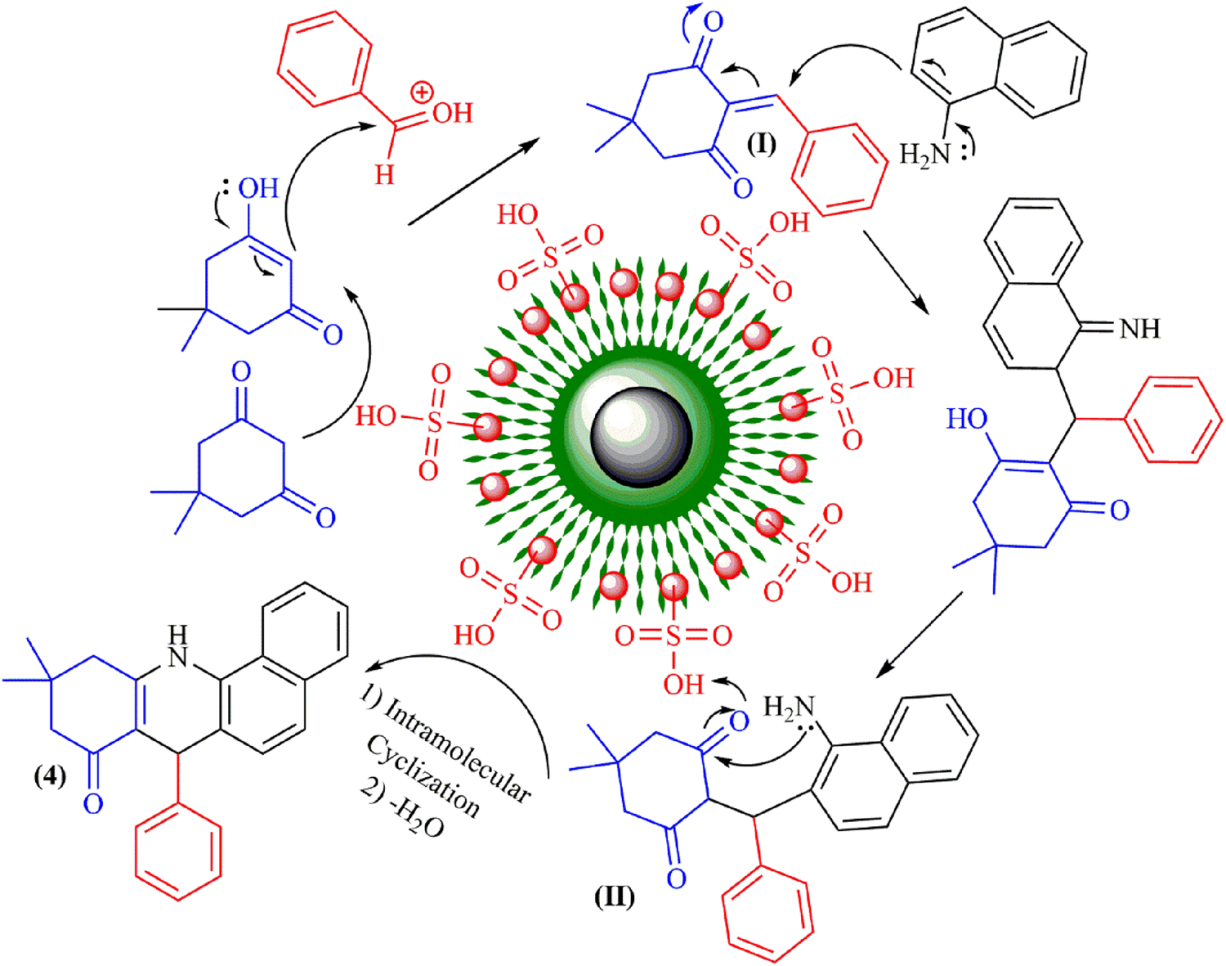
A proposed mechanism for the synthesis of benzo[*c*]acridin-8(9*H*)-one in the presence of Fe_3_O_4_@SiO_2_@TAD-G2-SO_3_H nanostructure.

A plausible mechanism is shown in Fig. 9. The carbonyl group of the aldehyde is initially protonated using the acidic catalyst. Subsequently, dimedone is enolized in acidic condition and undergoes condensation with activated aldehyde, leading to the formation of an intermediate (**I**). In the next step, the intermediate (**I**) is subjected to nucleophilic attack by 1-naphthylamine and followed by tautomerization, resulting in the production of structure (**II**). The intramolecular cyclization of compound (**II**) and elimination of water ultimately yields the benzoacridine (**4**).

The catalytic activity of Fe_3_O_4_@SiO_2_@TAD-G2-SO_3_H MNPs for the model reaction was also compared with that reported in the literature (Table 3). These data confirm that the Fe_3_O_4_@SiO_2_@TAD-G2-SO_3_H nanostructure is a suitable catalyst for synthesizing the desired corresponding product. The absence of the need for extraction with organic solvents not only simplifies the synthesis process but also reduces the environmental impact of the reaction. Overall, these results highlight the potential of this nanostructure as a green and efficient catalyst for other organic transformations.

**Table 3:** Comparison of catalytic activity of Fe_3_O_4_@SiO_2_@TAD-G2-SO_3_H with reported works for the synthesis of benzo[*c*]acridin-8(9*H*)-one derivatives.

Entry	Catalyst	Condition	Time (h)	Product (%)	[Ref]
1	[DSTMG][CCl_3_COO]	Neat, 85 °C, Extraction with CH_2_Cl_2_	0.25 h	**5b** (88)	^13^
2	[DSTMG][CF_3_COO]	Neat, 85 °C, Extraction with CH_2_Cl_2_	0.2 h	**5b** (90)	^13^
3	Succinimide-N-sulfonic acid	EtOH, 60 °C, Extraction with ethyl acetate	1.5 h	**5b** (95)	^18^
4	L-Proline	EtOH, rt, Extraction with ethyl acetate	2 h	**5b** (97)	^20^
5	KO_2_ and 18-crown-6	Toluene, rt, Extraction with CH_2_Cl_2_	10 h	**5b** (88)	^21^
6	Cu/MCM-41	EtOH, Reflux, Column chromatography	2 h	**5b** (92)	^22^
7	Fe_3_O_4_@SiO_2_@TAD-G2-SO_3_H	H_2_O/EtOH (1:1), 70 °C	3 h	**5b** (95)	This work

### Catalyst recovery and reuse

**Figure 10:**
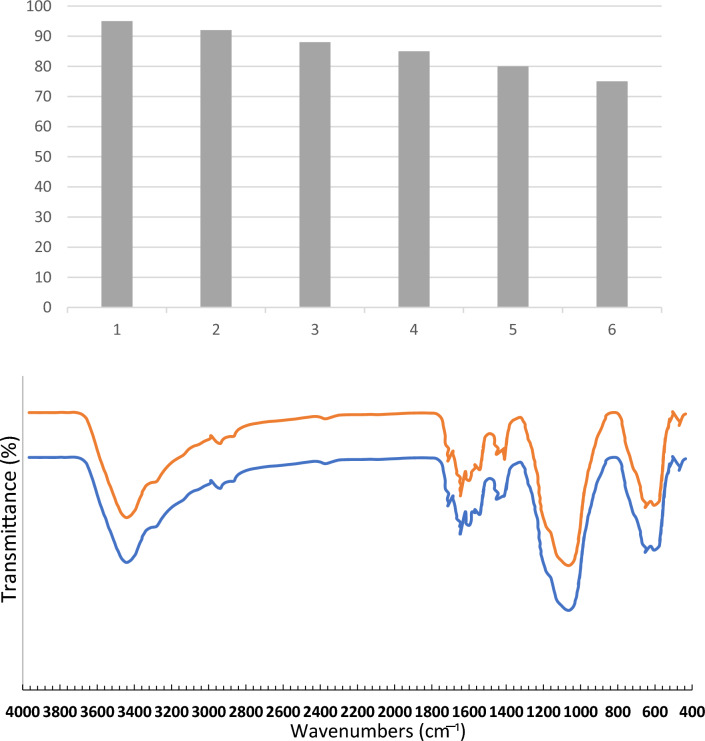
Reusability of the catalyst, and FT-IR spectra of the fresh and reused nanocatalyst.

The catalyst retrievability plays a crucial role in industrial applications, as well as in promoting environmentally friendly processes. Therefore, we conducted an investigation into the reusability of the Fe_3_O_4_@SiO_2_@TAD-G2-SO_3_H catalyst in a sequential reaction involving 4-chlorobenzaldehyde (1 mmol), 1-naphtylamine (1 mmol), and dimedone (1 mmol) in a H_2_O/EtOH (1:1) mixture at 70 °C. After the reaction was completed, the resulting mixture solidified. To dilute it, 10 mL of hot EtOH was added. The catalyst was easily separated using an external magnet, washed with hot EtOH, dried under vacuum, and reused in the next reaction. Each run allowed for the nearly complete recovery of the catalyst, with up to 95% being obtained. The reused catalyst exhibits high efficiency in the synthesis of benzoacridine, as depicted in Fig. 10. The stability of the catalyst is further considered by the FT-IR spectra of the reused catalyst (Fig. 10), which proves the stability of catalyst in reaction condition.

## Conclusions

A solid acid catalyst (Fe_3_O_4_@SiO_2_@TAD-G2-SO_3_H) which can be easily retrieved using magnets, was successfully synthesized and characterized. This catalyst was created by growing triazine dendrimer on silica-coated magnetic nanoparticles, while also immobilizing sulfonic acid groups. The prepared dendritic nanostructure (Fe_3_O_4_@SiO_2_@TAD-G2-SO_3_H) efficiently catalyzed the synthesis of benzo[*c*]acridin-8(9*H*)-one and benzo[*h*]indeno[1,2-*b*]quinoline-8-one derivatives in excellent yields (90% to 96%). Grafting of dendrimer increases the active sites on Fe_3_O_4_ nanoparticles and decreases their agglomeration. The catalyst can be easily separated from the reaction mixture by employing a magnetic field, allowing for its recycling up to five times with slight loss in its activity (10%). Other advantages of this protocol include the use of aqueous ethanol as a mild reaction media, operational simplicity, high to excellent yields, high atom-economy, easy separation of the catalyst, reusability, and elimination of the need for tedious column chromatography during product isolation and purification. These aspects align with the principles of green and sustainable chemistry, which aim to design chemical products and processes that reduce or eliminate hazardous substances and waste generation.

## Data Availability

All data generated or analysed during this study are included in this article.

## References

[1] Ganem B (2009). Strategies for innovation in multicomponent reaction design. Acc. Chem. Res..

[2] Toure BB, Hall DG (2009). Natural product synthesis using multicomponent reaction strategies. Chem. Rev..

[3] Khurana JM, Maikap GC, Mehta S (1990). Reductive coupling of geminal dichlorides by iron (II) oxalate dihydrate. Synthesis.

[4] Gamage SA (1999). Structure—activity relationships for substituted bis (acridine-4-carboxamides): A new class of anticancer agents. J. Med. Chem..

[5] Yang P, Yang Q, Qian X, Tong L, Li X (2006). Isoquino [4, 5-bc] acridines: Design, synthesis and evaluation of DNA binding, anti-tumor and DNA photo-damaging ability. J. Photochem. Photobiol. B Biol..

[6] Girault S (2000). Antimalarial, antitrypanosomal, and antileishmanial activities and cytotoxicity of bis (9-amino-6-chloro-2-methoxyacridines): Influence of the linker. J. Med. Chem..

[7] Jamalian A (2011). Synthesis, cytotoxicity and calcium antagonist activity of novel imidazolyl derivatives of 1, 8-acridinediones. J. Iran. Chem. Soc..

[8] Sondhi SM (2010). Synthesis, anti-inflammatory and anticancer activity evaluation of some novel acridine derivatives. Eur. J. Med. Chem..

[9] Denny WA (2002). Acridine derivatives as chemotherapeutic agents. Current Med. Chem..

[10] Tu S-J (2006). An efficient one-pot, three-component synthesis of indeno [1, 2-b] quinoline-9, 11 (6H, 10H)-dione, acridine-1, 8 (2H, 5H)-dione and quinoline-3-carbonitrile derivatives from enaminones. Organ. Biomol. Chem..

[11] Deady LW (2000). Synthesis and antitumor activity of some indeno [1, 2-b] quinoline-based bis carboxamides. Bioorgan. Med. Chem..

[12] Rampa A (2000). Acetylcholinesterase inhibitors for potential use in Alzheimer's disease: molecular modeling, synthesis and kinetic evaluation of 11H-indeno-[1, 2-b]-quinolin-10-ylamine derivatives. Bioorgan. Med. Chem..

[13] Dutta AK, Gogoi P, Saikia S, Borah R (2017). N, N-disulfo-1, 1, 3, 3-tetramethylguanidinium carboxylate ionic liquids as reusable homogeneous catalysts for multicomponent synthesis of tetrahydrobenzo [a] xanthene and tetrahydrobenzo [a] acridine derivatives. J. Mol. Liquids.

[14] Zang H, Zhang Y, Zang Y, Cheng B-W (2010). An efficient ultrasound-promoted method for the one-pot synthesis of 7, 10, 11, 12-tetrahydrobenzo [c] acridin-8 (9H)-one derivatives. Ultrason. Sonochem..

[15] Wang X-S (2006). A clean procedure for synthesis of benzo [c] acridine derivatives: reaction of N-arylidenenaphthalen-1-amine with 5, 5-dimethyl-1, 3-cyclohexadione in aqueous medium. Arkivoc.

[16] Mamaghani M, Larghani TH (2012). Ultrasound promoted one-pot three-component synthesis of novel 7-aryl-8 H-benzo [h] indeno [1, 2-b] quinolin-8-ones under solvent-free conditions. J. Chem. Res..

[17] Mansoor SS, Ghashang M, Aswin K (2015). Facile one-pot synthesis of a novel series of 7-aryl-8 H-benzo [h] indeno [1, 2-b] quinoline-8-one derivatives catalyzed by tribromomelamine. Res. Chem. Intermed..

[18] Ghashang M, Mansoor SS, Aswin K (2017). Succinimide-N-sulfonic acid: An efficient and recyclable catalyst for the one-pot synthesis of tetrahydrobenzo [c] acridine-8 (7H)-one derivatives. J. Saudi Chem. Soc..

[19] Maleki A, Nooraie Yeganeh N (2017). Facile one-pot synthesis of a series of 7-aryl-8H-benzo [h] indeno [1, 2-b] quinoline-8-one derivatives catalyzed by cellulose-based magnetic nanocomposite. Appl. Organomet. Chem..

[20] Heravi MRP, Aghamohammadi P (2012). l-Proline-catalysed one-pot synthesis of tetrahydrobenzo [c] acridin-8 (7H)-ones at room temperature. Comptes. Rendus. Chimie.

[21] Gajaganti S, Singh S (2017). Superoxide Ion Prompted One Pot Multicomponent Synthesis of 1, 4-dihydropyridine derivatives. Mater. Today Proceed..

[22] Dhengale SD (2023). An efficient and convenient heterogeneous Cu/MCM-41 catalyst for the synthesis of 7, 10, 11, 12-tetrahydrobenzo [c] acridin-8 (9H)-one derivatives. Res. Chem. Intermed..

[23] Arshadi M, Abdolmaleki M, Eskandarloo H, Azizi M, Abbaspourrad A (2018). Synthesis of highly monodispersed, stable, and spherical NZVI of 20–30 nm on filter paper for the removal of phosphate from wastewater: batch and column study. ACS Sustain. Chem. Eng..

[24] Svenson S, Tomalia DA (2012). Dendrimers in biomedical applications—reflections on the field. Adv. Drug Deliv. Rev..

[25] Eskandarian L, Pajootan E, Arami M (2014). Novel super adsorbent molecules, carbon nanotubes modified by dendrimer miniature structure, for the removal of trace organic dyes. Ind. Eng. Chem. Res..

[26] Sajid M, Nazal MK, Baig N, Osman AM (2018). Removal of heavy metals and organic pollutants from water using dendritic polymers based adsorbents: A critical review. Sep. Purif. Technol..

[27] Kuzin A (2023). Bridging the gap: harnessing liquid nanomachine know-how for tackling harmful airborne particulates. Nanoscale.

[28] Mohan B (2023). Nanomaterials for miRNA detection: The hybridization chain reaction strategy. Sens. Diagn..

[29] Shylesh S, Schünemann V, Thiel WR (2010). Magnetically separable nanocatalysts: Bridges between homogeneous and heterogeneous catalysis. Angew. Chemie Int. Edition.

[30] Hedayati K, Kord M, Goodarzi M, Ghanbari D, Gharigh S (2017). Photo-catalyst and magnetic nanocomposites: hydrothermal preparation of core–shell Fe 3 O 4@ PbS for photo-degradation of toxic dyes. J. Mater. Sci. Mater. Electron..

[31] Yen C-H, Lien H-L, Chung J-S, Yeh H-D (2017). Adsorption of precious metals in water by dendrimer modified magnetic nanoparticles. J. Hazardous Mater..

[32] Kim K-J, Park J-W (2017). Stability and reusability of amine-functionalized magnetic-cored dendrimer for heavy metal adsorption. J. Mater. Sci..

[33] Ahangarani-Farahani R, Bodaghifard MA (2022). A polyethanolamine nanodendrimer as a magnetic hybrid material for fast adsorption of heavy metal contaminants. J. Mater. Sci. Mater. Electron..

[34] Ahangarani-Farahani R, Bodaghifard MA, Asadbegi S (2022). Magnetic triazine-based dendrimer as a versatile nanocarrier for efficient antiviral drugs delivery. Sci. Rep..

[35] Bodaghifard A, Faraki MZ, Karimi RA (2016). Mild synthesis of mono-, bis-and tris 1, 2-dihydrobenzo [4, 5] imidazo [1, 2-a] pyrimidine derivatives using alkyl disulfamic acid functionalized magnetic nanoparticles. Curr. Organ. Chem..

[36] Bodaghifard MA (2019). Palladium-melamine complex anchored on magnetic nanoparticles: A novel promoter for CC cross coupling reaction. J. Organ. Chem..

[37] Asadbegi S, Bodaghifard MA, Mobinikhaledi A (2020). Poly N, N-dimethylaniline-formaldehyde supported on silica-coated magnetic nanoparticles: A novel and retrievable catalyst for green synthesis of 2-amino-3-cyanopyridines. Res. Chem. Intermed..

[38] Hamidinasab M, Bodaghifard MA, Mobinikhaledi A (2020). Green synthesis of 1H-pyrazolo [1, 2-b] phthalazine-2-carbonitrile derivatives using a new bifunctional base–ionic liquid hybrid magnetic nanocatalyst. Appl. Organ. Chem..

[39] Gheni SA, Hmood HM, Ahmed SM, Mohammed MH (2023). Optimization for kinetic model of oxidative desulfurization of sour naphtha over a natural base zeolite catalyst in a three phase oscillatory baffled reactor. J. Petrol. Res. Studies.

[40] Mohammed AE (2024). Agricultural waste-based microporous catalysts for oxidative desulfurization of highly sour heavy gas oil. Diamond. Related Mater..

[41] Bodaghifard MA, Hamidinasab M, Ahadi N (2018). Recent advances in the preparation and application of organic–inorganic hybrid magnetic nanocatalysts on multicomponent reactions. Curr. Organ. Chem..

[42] Geißler D, Nirmalananthan-Budau N, Scholtz L, Tavernaro I, Resch-Genger U (2021). Analyzing the surface of functional nanomaterials—how to quantify the total and derivatizable number of functional groups and ligands. Microchimica Acta.

[43] Kainz QM, Reiser O (2014). Polymer-and dendrimer-coated magnetic nanoparticles as versatile supports for catalysts, scavengers, and reagents. Accounts Chem. Res..

[44] Bahrami K, Arabi MS (2016). Copper immobilized ferromagnetic nanoparticle triazine dendrimer (FMNP@ TD–Cu (ii))-catalyzed regioselective synthesis of 1, 4-disubstituted 1, 2, 3-triazoles. New J. Chem..

[45] Pan S, Yan S, Osako T, Uozumi Y (2017). Batch and continuous-flow Huisgen 1, 3-dipolar cycloadditions with an amphiphilic resin-supported triazine-based polyethyleneamine dendrimer copper catalyst. ACS Sustain. Chem. Eng..

[46] Niakan M, Asadi Z, Masteri-Farahani M (2020). Fe (III)-salen complex supported on dendrimer functionalized magnetite nanoparticles as a highly active and selective catalyst for the green oxidation of sulfides. J. Phys. Chem. Solids.

[47] Saberi D, Hashemi H, Ghanaatzadeh N, Moghadam M, Niknam K (2020). Ruthenium/dendrimer complex immobilized on silica-functionalized magnetite nanoparticles catalyzed oxidation of stilbenes to benzil derivatives at room temperature. Appl. Organomet. Chem..

[48] Karimi S, Shekaari H, Halimehjani AZ, Niakan M (2020). Solvent-free production of 5-hydroxymethylfurfural from deep eutectic substrate reaction mixtures over a magnetically recoverable solid acid catalyst. ACS Sustain. Chem. Eng..

[49] Ahadi N, Mobinikhaledi A, Bodaghifard MA (2020). One-pot synthesis of 1, 4-dihydropyridines and N-arylquinolines in the presence of copper complex stabilized on MnFe2O4 (MFO) as a novel organic–inorganic hybrid material and magnetically retrievable catalyst. Appl. Organomet. Chem..

[50] Mohan B (2018). Design and synthesis of two armed molecular receptor for recognition of Gd3+ metal ion and its computational study. Appl. Organomet. Chem..

[51] Mohan B (2018). Selectivity for La3+ ion by synthesized 4-((5-methylfuran-2-yl) methylene) hydrazono) methyl) phenol receptor and its spectral analysis. Spectroch. Acta Part A Mol. Biomol. Spectrosc..

[52] Mohan B, Ma S, Kumar S, Yang Y, Ren P (2023). Tactile sensors: Hydroxyl decorated silver metal-organic frameworks for detecting Cr2O72–, MnO4–, Humic Acid, and Fe3+ Ions. ACS Appl. Mater. Interfaces.

[53] Zhou Q (2021). Magnetic polyamidoamine dendrimers for magnetic separation and sensitive determination of organochlorine pesticides from water samples by high-performance liquid chromatography. J. Environ. Sci..

[54] Asadbegi S, Bodaghifard MA, Alimohammadi E, Ahangarani-Farahani R (2018). Immobilization of palladium on modified nanoparticles and its catalytic properties on mizoroki-heck reaction. ChemistrySelect.

